# Construction of a new tool for predicting cancer-specific survival in papillary thyroid cancer patients who have not received surgery

**DOI:** 10.3389/fendo.2024.1417528

**Published:** 2024-08-16

**Authors:** Sanjun Chen, Yanmei Tan, Xinping Huang, Yanfei Tan

**Affiliations:** ^1^ Department of Pain, The First People’s Hospital of Chenzhou, The First Affiliated Hospital of Xiangnan University, Chenzhou, Hunan, China; ^2^ School of Basic Medicine, Xiangnan University, Chenzhou, Hunan, China; ^3^ Department of Metabolism and Endocrinology, The First People’s Hospital of Chenzhou, The First Affiliated Hospital of Xiangnan University, Chenzhou, Hunan, China

**Keywords:** papillary thyroid cancer, non-operative, PSM, nomogram, SEER

## Abstract

**Background:**

The prevalence of papillary thyroid cancer is gradually increasing and the trend of youthfulness is obvious. Some patients may not be able to undergo surgery, which is the mainstay of treatment, due to physical or financial reasons. Therefore, the prediction of cancer-specific survival (CSS) in patients with non-operated papillary thyroid cancer is necessary.

**Methods:**

Patients’ demographic and clinical information was extracted from the Surveillance, Epidemiology, and End Results database. SPSS software was used to perform Cox regression analyses as well as propensity score matching analyses. R software was used to construct and validate the nomogram. X-tile software was used to select the best cutoff point for patient risk stratification.

**Results:**

A total of 1319 patients were included in this retrospective study. After Cox regression analysis, age, grade, T stage, M stage, radiotherapy, and chemotherapy were used to construct the nomogram. C-index, calibration curves, and receiver operating characteristic curves all verified the high predictive accuracy of the nomogram. The decision curve analysis demonstrated that patients could gain clinical benefit from this predictive model. Survival curve analysis after propensity score matching demonstrated the positive effects of radiotherapy on CSS in non-operated patients.

**Conclusion:**

Our retrospective study successfully established a nomogram that accurately predicts CSS in patients with non-operated papillary thyroid cancer and demonstrated that radiotherapy for operated patients can still help improve prognosis. These findings can help clinicians make better choices.

## Introduction

In recent years, the incidence of papillary thyroid cancer has been increasing every year (1). According to the 2020 WHO Cancer Incidence and Mortality Database, thyroid malignant tumors rank as the ninth most common cancer worldwide (2). Females show a higher prevalence, with a ratio of female patients to male patients of approximately 3:1 (3, 4). Thyroid cancer can be categorized into many histological types with great prognostic differences, including papillary thyroid cancer, medullary carcinoma, and interstitial thyroid carcinoma, of which the most common malignant type is papillary thyroid cancer, accounting for about 80% of cases, so this study discusses papillary thyroid cancer (3). Thyroid cancer, as the most common malignant tumor in adolescents and adults aged 16-33, poses a serious threat to patients’ lives and health (5).

Many malignant thyroid tumors require surgical removal (6, 7). The extent of the surgery depends on the size and spread of the tumor and includes the removal of a lobe or the entire thyroid gland. For patients with high-risk thyroid cancer, postoperative radiation therapy can reduce the risk of recurrence, and this is usually done after total thyroidectomy. Radiation therapy can also be used to manage residual thyroid tissue after surgery or local recurrence of thyroid cancer (8, 9). This helps to reduce the size of the tumor and control the disease. While surgical treatment is often considered the first option for thyroid cancer patients, it is undeniable that surgical treatment may not be appropriate for all thyroid cancer patients. First, those with thyroid cancer with fatal disease may not be candidates for surgical treatment because they cannot tolerate surgery. Secondly, the cost of surgical treatment may not be affordable for all thyroid cancer patients, who may choose to decline surgery for financial reasons. Finally, some thyroid cancer patients may have lost the opportunity for surgery, and thus clinicians may not recommend surgery. Therefore, we need to pay more attention to those thyroid cancer patients who are not treated with surgery.

Nomogram is an emerging event prediction model that has been widely used to predict the prognosis of patients with a variety of cancers (10, 11). It can give an accurate prognostic evaluation based on different disease-related information for each individual. Therefore, for the first time, we have developed a nomogram that can predict the prognosis of patients with papillary thyroid cancer who have not undergone surgery, which can help clinicians provide the best treatment options for patients.

## Method

### Data collection and demographics

Patient data from the retrospective study came from the National Cancer Institute’s Surveillance, Epidemiology, and End Results (SEER) database, which covers cancer information for 28 percent of the total US population. Patient consent is not required because our study is retrospective and does not involve patients’ personally identifiable information.

The inclusion and exclusion criteria for extracting and screening data from the SEER database were as follows: The inclusion criteria: (1) Primary papillary thyroid cancer (International Classification of Disease for Oncology histopathology codes: 8050, 8260, 8340, 8341, 8344, 8347); (2) For patients who refuse to undergo the surgery or who are not recommended by their clinician to undergo the surgery; (3) Diagnosed between 2010 and 2015. The exclusion criteria: (1) Incomplete information regarding patient ID and survival status; (2) Lack of information on follow-up information; (3) Carcinoma *in situ*. Clinical pathological data and demographic data were extracted from patients including age, sex, race, tumor grade, TNM stage, radiotherapy, and chemotherapy status. Outcome factors include survival time and cause-specific death.

### Statistical analysis

The total cohort was randomly assigned into a training set and a validation set in a ratio of 7:3. A Cox proportional hazards regression model was used for multivariate analysis to determine independent prognostic factors for thyroid cancer patients without surgery. The training cohort involved employing univariate and multivariate Cox regression analyses to identify risk variables affecting postoperative cancer-specific survival (CSS). Using R software, a predictive model for postoperative CSS was then established from the results of the multivariate Cox regression analysis. Additionally, receiver operating characteristic curves (ROC) were generated, and the area under the curve (AUC) was computed to evaluate the model’s discriminatory capacity. Calibration curves and decision curve analysis (DCA) were developed for 3, 5, and 8 years to assess predictive accuracy and clinical utility. X-tile software was used to select the best cutoff point for the nomogram score. Based on this score cutoff point patients were categorized into three risk strata and Kaplan-Meier survival curves were plotted. Finally, patients were propensity score matched (PSM) in a 1:1 ratio based on radiotherapy, with a caliper of 0.01, and Kaplan-Meier survival curves were plotted to assess outcome differences. All statistical analysis and charting were carried out by using SPSS (25.0) and R software version 4.3.1. A two-tailed P<0.05 was considered statistically significant.

## Results

### Inclusion of patients

A total of 200950 patients with primary papillary thyroid cancer diagnosed between 2010 and 2015 were identified from the SEER database. Among them, 9072 patients were included because of lack of surgery. After excluding patients whose ID, survival information, and variables were unknown, 1319 patients were finally included in our study. Demographic and clinical characteristics of the patients are shown in **Table 1**.

**Table 1:** Clinical and pathological characteristics of patients with non-operative papillary thyroid cancer.

Characteristic	Group			p-value
	Overall N = 1,319	Training cohort N = 924	Validation cohort N = 395	
Age				0.90
≤60	491 (37.23%)	343 (37.12%)	148 (37.47%)	
>60	828 (62.77%)	581 (62.88%)	247 (62.53%)	
Sex				0.49
Male	479 (36.32%)	330 (35.71%)	149 (37.72%)	
Female	840 (63.68%)	594 (64.29%)	246 (62.28%)	
Race				0.20
Black	109 (8.26%)	78 (8.44%)	31 (7.85%)	
White	1,002 (75.97%)	690 (74.68%)	312 (78.99%)	
Other	208 (15.77%)	156 (16.88%)	52 (13.16%)	
Grade				0.41
Grade I	19 (1.44%)	13 (1.41%)	6 (1.52%)	
Grade II	7 (0.53%)	6 (0.65%)	1 (0.25%)	
Grade III	51 (3.87%)	30 (3.25%)	21 (5.32%)	
Grade IV	289 (21.91%)	201 (21.75%)	88 (22.28%)	
Unkown	953 (72.25%)	674 (72.94%)	279 (70.63%)	
T stage				0.61
T1	456 (34.57%)	327 (35.39%)	129 (32.66%)	
T2	248 (18.80%)	177 (19.16%)	71 (17.97%)	
T3	134 (10.16%)	90 (9.74%)	44 (11.14%)	
T4	481 (36.47%)	330 (35.71%)	151 (38.23%)	
N stage				0.16
N0	888 (67.32%)	633 (68.51%)	255 (64.56%)	
N1	431 (32.68%)	291 (31.49%)	140 (35.44%)	
M stage				0.66
M0	979 (74.22%)	689 (74.57%)	290 (73.42%)	
M1	340 (25.78%)	235 (25.43%)	105 (26.58%)	
Radiotherapy				0.74
No	1,112 (84.31%)	781 (84.52%)	331 (83.80%)	
Yes	207 (15.69%)	143 (15.48%)	64 (16.20%)	
Chemotherapy				0.93
No	1,167 (88.48%)	818 (88.53%)	349 (88.35%)	
Yes	152 (11.52%)	106 (11.47%)	46 (11.65%)	

### Independent predictors of cancer-specific survival

We conducted univariable Cox regression analyses to recognize the factors significantly associated with CSS of thyroid cancer patients without surgery. As shown in **Table 2**, age, race, T stage, N stage, M stage, radiotherapy, chemotherapy, and grade were significantly associated with poor CSS (P<0.05).

**Table 2:** Analysis of univariate and multivariate Cox regression in patients with non-operative papillary thyroid cancer.

Characteristics	Univariate analysis		Multivariate analysis	
	HR (95% CI)	P value	HR (95% CI)	P value
Age				
≤60	Reference		Reference	
>60	0.241 (0.181-0.322)	<0.001	0.528 (0.391-0.714)	<0.001
Sex				
Male	Reference			
Female	1.117 (0.901-1.385)	0.314		
Race				
Black	Reference		Reference	
White	1.542 (1.026-2.319)	0.037	1.273 (0.842-1.923)	0.252
Other	1.066 (0.800-1.421)	0.661	1.157 (0.864-1.550)	0.328
Grade				
Grade I	Reference		Reference	
Grade II	0.302 (0.042-2.161)	0.233	0.317 (0.044-2.275)	0.253
Grade III	7.275 (2.673-19.799)	<0.001	2.696 (0.974-7.461)	0.056
Grade IV	7.901 (5.088-12.269)	<0.001	3.191 (2.005-5.080)	<0.001
Unknown	12.200 (9.504-15.659)	<0.001	4.341 (3.138-6.006)	<0.001
T stage				
T1	Reference		Reference	
T2	0.029 (0.017-0.048)	<0.001	0.077 (0.044-0.137)	<0.001
T3	0.093 (0.062-0.138)	<0.001	0.236 (0.148-0.377)	<0.001
T4	0.276 (0.192-0.396)	<0.001	0.601 (0.395-0.916)	0.018
N stage				
N0	Reference		Reference	
N1	0.240 (0.194-0.297)	<0.001	0.868 (0.683-1.104)	0.248
M stage				
M0	Reference		Reference	
M1	0.144 (0.116-0.179)	<0.001	0.449 (0.354-0.569)	<0.001
Radiotherapy				
No	Reference		Reference	
Yes	0.421 (0.334-0.530)	<0.001	1.778 (1.379-2.291)	<0.001
Chemotherapy				
No	Reference		Reference	
Yes	0.381 (0.297-0.490)	<0.001	1.752 (1.319-2.328)	<0.001

We conducted a multivariable Cox regression analysis, including the significant variables by univariate analyses, to explore independent predictors of the CSS. As shown in **Table 2**, age, grade, T stage, M stage, radiotherapy, and chemotherapy were independent predictors of poor CSS (P<0.05).

### Construction and validation of a nomogram

**Figure 1:**
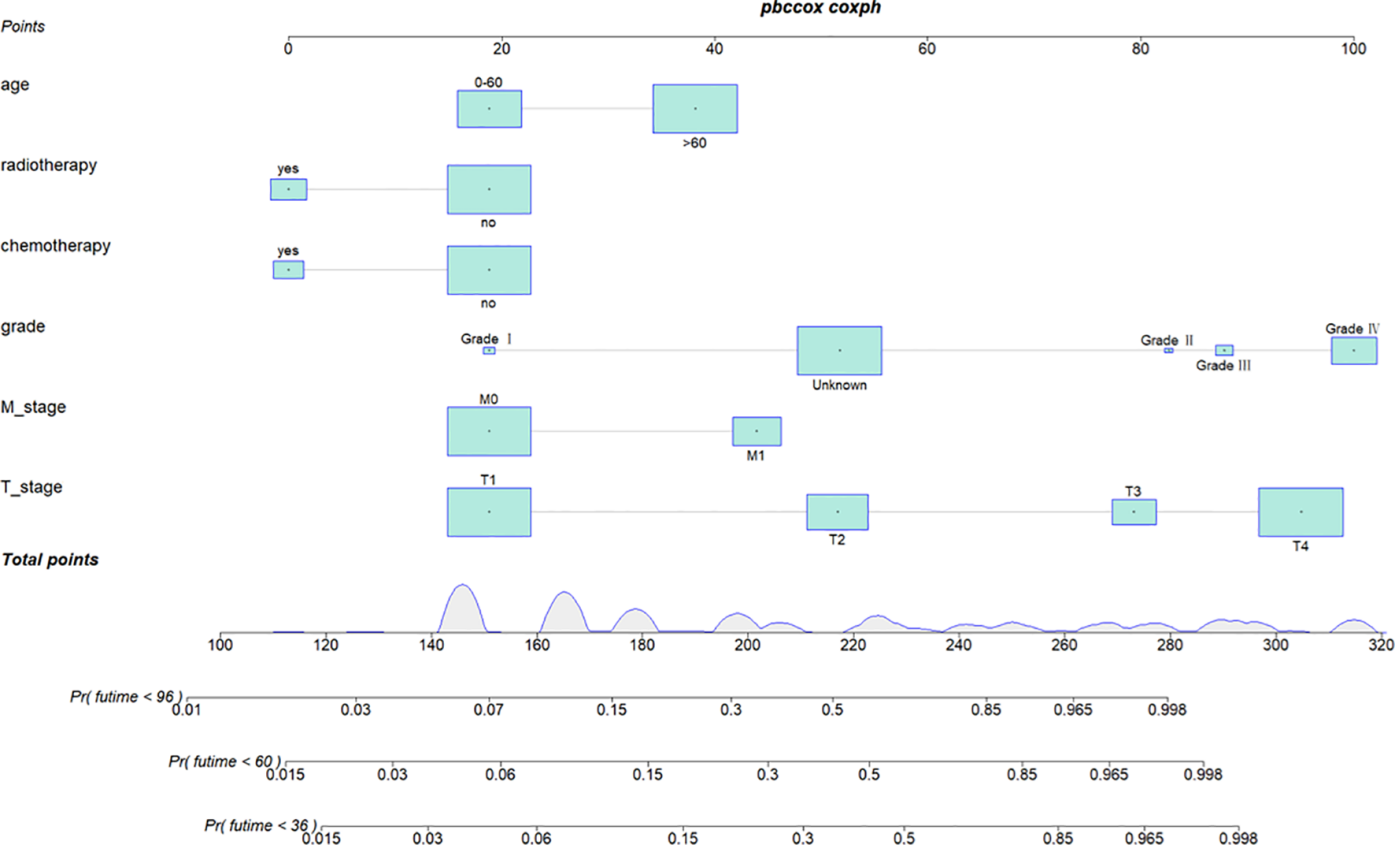
Nomogram for predicting 3-year, 5-year, and 8-year cancer-specific survival of patients with non-operative papillary thyroid cancer.

**Figure 2:**
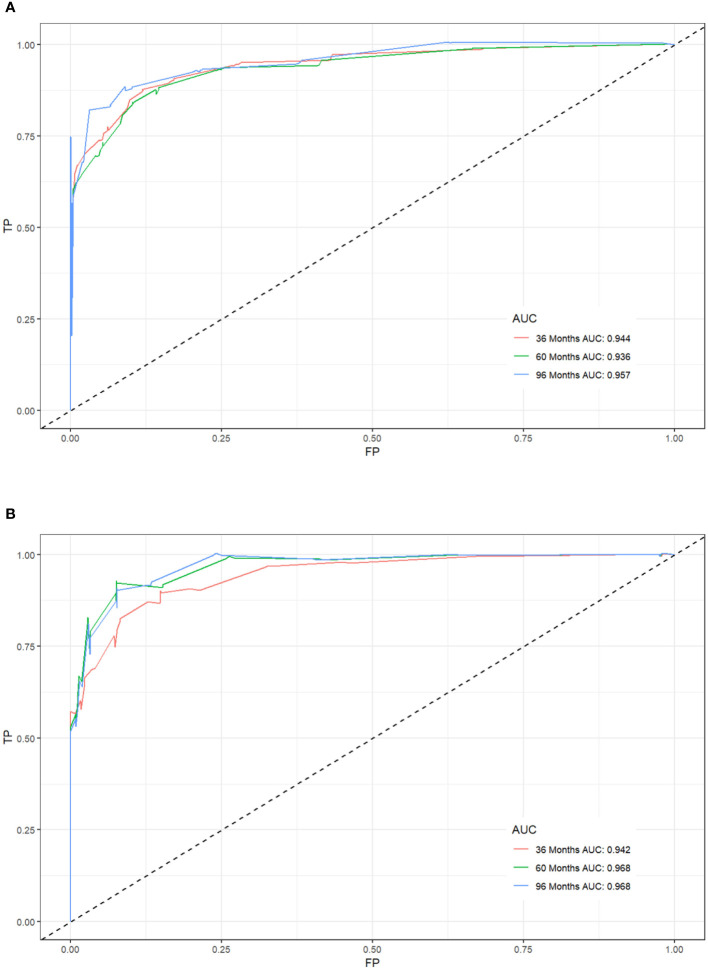
Receiver operating characteristic (ROC) curves for postoperative cancer-specific survival prediction of patients with non-operative papillary thyroid cancer. **(A)** Training cohort, **(B)** Validation cohort.

**Figure 3:**
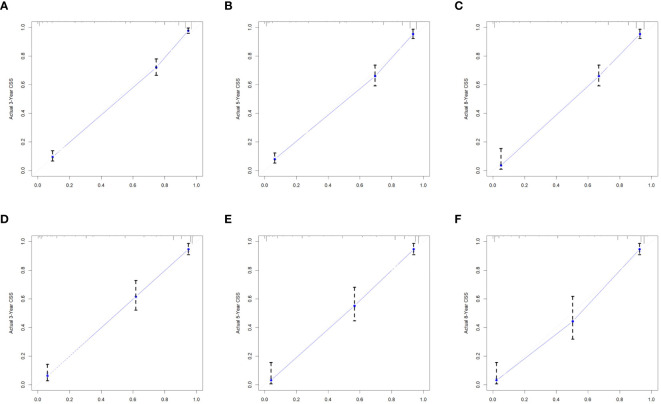
Calibration of the nomogram model in the training cohort **(A-C)**, and validation cohort **(D-F)**.

**Figure 4:**
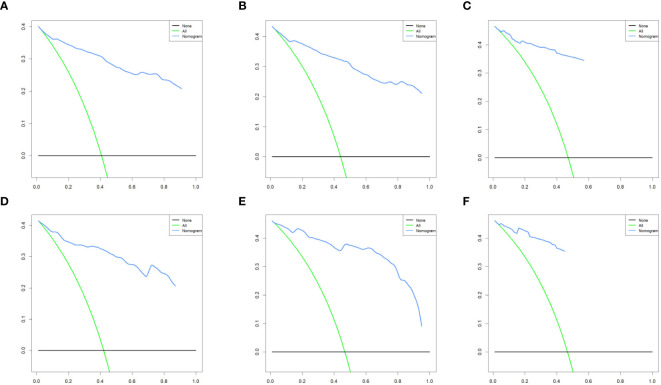
Decision curve analysis of the nomogram model in the training cohort **(A-C)**, and validation cohort **(D-F)**.

**Figure 5:**
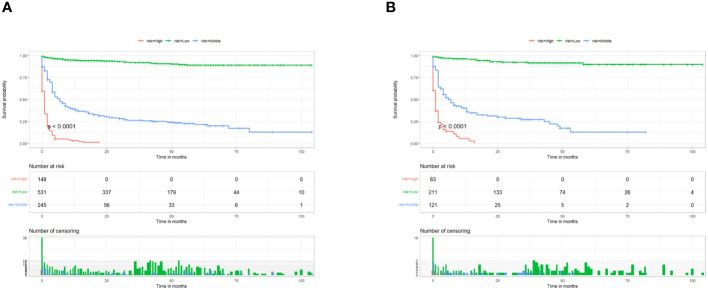
Kaplan–Meier survival analyses were performed to compare postoperative cancer-specific survival in the low risk, middle risk, and high risk subgroups of all patients in the training cohort **(A)** and validation cohort **(B)**.

We combined four independent prognostic factors for CSS and developed a nomogram for predicting the CSS of patients (**Figure 1**). The C-index in the training and validation queues are 0.885 and 0.874, respectively. The AUC values at 3, 5, and 8 years were 0.944, 0.936, and 0.957 in the training cohort and 0.942, 0.968, and 0.968 in the validation cohort (**Figure 2**). The C-index and AUC values indicate robust predictive performance in both cohorts. The calibration curve closely mirrors the 45° line, signifying strong agreement between the nomogram’s predictions and actual outcomes (**Figure 3**). In both training and validation cohorts, the DCA underscores the nomogram’s valuable clinical utility for predicting CSS in thyroid cancer patients without surgery (**Figure 4**). Kaplan-Meier survival curves demonstrated significant differences in survival outcomes for patients in different risk strata (**Figure 5**).

### PSM and survival curve based on radiotherapy

**Figure 6:**
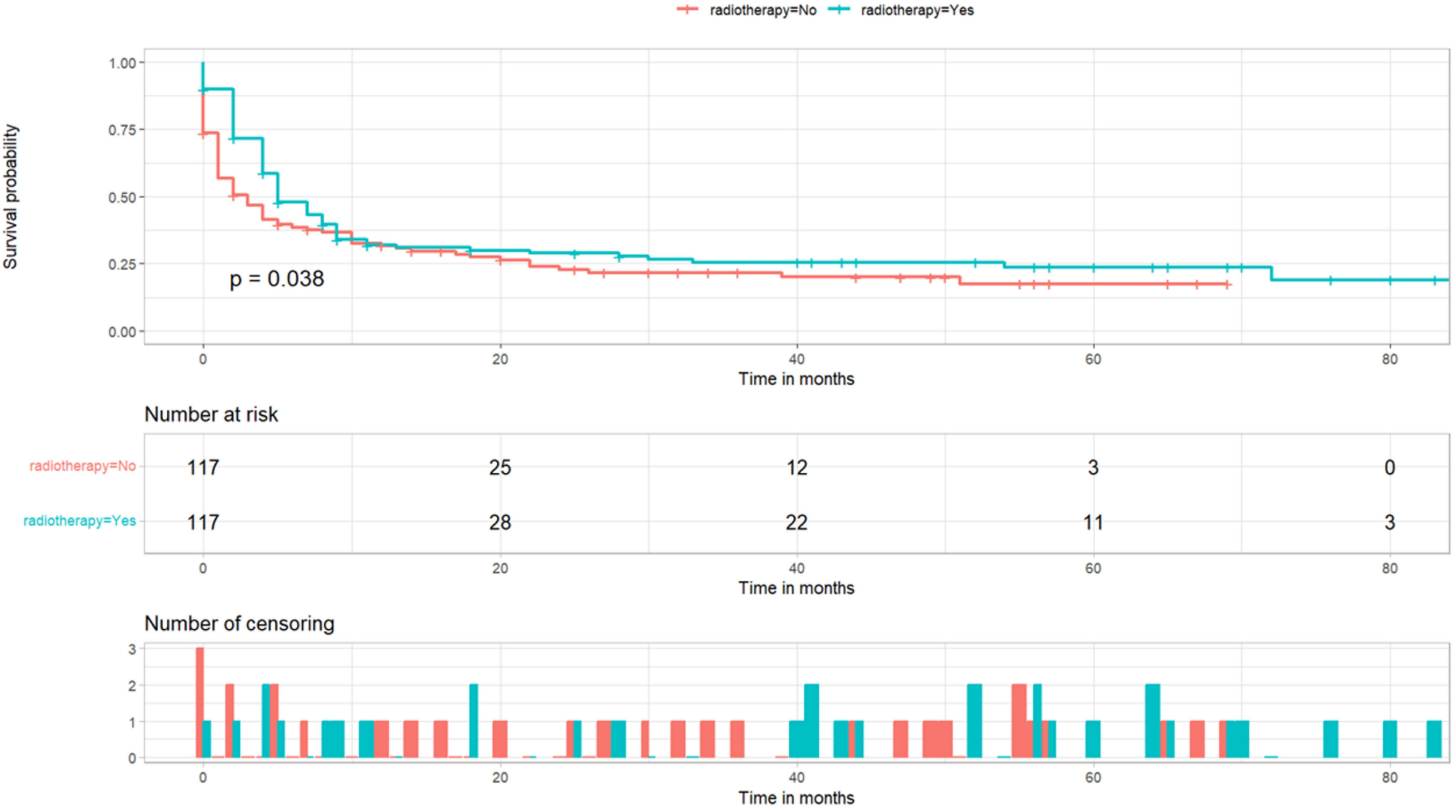
Kaplan–Meier survival analyses were performed to compare postoperative cancer-specific survival for patients receiving radiotherapy and those not receiving radiotherapy after PSM analysis.

A 1:1 PSM analysis was performed on the training cohort based on whether they received radiotherapy. It can be seen that after PSM analysis, the baseline information of patients in the matched cohort reached equilibrium (P>0.05) (**Table 3**). The Kaplan-Meier survival curves instructed that unoperated patients with thyroid cancer who received radiotherapy could produce a trend of significant change in the survival outcome (P< 0.05), as shown in **Figure 6**.

**Table 3:** Baseline table of the population after PSM analysis using radiotherapy receipt as a subgrouping criterion.

Characteristic	Radiotherapy			p-value
	Overall N = 234	No N = 117	Yes N = 117	
Age				0.20
≤60	48 (20.51%)	20 (17.09%)	28 (23.93%)	
>60	186 (79.49%)	97 (82.91%)	89 (76.07%)	
Sex				0.23
Male	95 (40.60%)	43 (36.75%)	52 (44.44%)	
Female	139 (59.40%)	74 (63.25%)	65 (55.56%)	
Race				0.35
Black	27 (11.54%)	13 (11.11%)	14 (11.97%)	
White	171 (73.08%)	82 (70.09%)	89 (76.07%)	
Other	36 (15.38%)	22 (18.80%)	14 (11.97%)	
Grade				0.36
Grade I	2 (0.85%)	0 (0.00%)	2 (1.71%)	
Grade II	3 (1.28%)	1 (0.85%)	2 (1.71%)	
Grade III	12 (5.13%)	8 (6.84%)	4 (3.42%)	
Grade IV	124 (52.99%)	58 (49.57%)	66 (56.41%)	
Unknown	93 (39.74%)	50 (42.74%)	43 (36.75%)	
T stage				0.46
T1	14 (5.98%)	8 (6.84%)	6 (5.13%)	
T2	11 (4.70%)	3 (2.56%)	8 (6.84%)	
T3	14 (5.98%)	7 (5.98%)	7 (5.98%)	
T4	195 (83.33%)	99 (84.62%)	96 (82.05%)	
N stage				0.60
N0	98 (41.88%)	47 (40.17%)	51 (43.59%)	
N1	136 (58.12%)	70 (59.83%)	66 (56.41%)	
M stage				0.90
M0	103 (44.02%)	51 (43.59%)	52 (44.44%)	
M1	131 (55.98%)	66 (56.41%)	65 (55.56%)	
Chemotherapy				0.89
No	155 (66.24%)	78 (66.67%)	77 (65.81%)	
Yes	79 (33.76%)	39 (33.33%)	40 (34.19%)	

## Discussion

In this study, we successfully developed a nomogram that can predict the prognosis of unoperated papillary thyroid cancer patients from the clinical information of the patients, and this nomogram was shown to have a good predictive effect in both the training cohort and the validation cohort. We found that the prognosis of patients with unoperated papillary thyroid cancer was correlated with age, grade, T stage, M stage, radiotherapy, and chemotherapy. In addition, we emphasize that radiotherapy remains an independent risk factor for patient survival after PSM analysis, demonstrating that receiving radiotherapy if a patient fails to undergo surgery remains a positive influence on long-term survival.

Age as one of the major high-risk predisposing factors for thyroid is also recognized as a factor affecting the prognostic survival of patients. A retrospective study noted that age AJCC/TNM staging showed consistency (12). This means that older patients are more likely to receive a late diagnosis of the tumor as well as a poorer prognosis compared to younger patients. Interestingly, our study points out that although gender is an important risk factor for thyroid cancer incidence, gender does not seem to affect patient prognosis. In a systematic review, researchers noted that male patients with papillary thyroid cancer were significantly more likely to develop lymph node metastases than female patients (13). However, there is a lack of reports demonstrating the correlation between gender and survival in thyroid cancer patients (14). This question still needs to be confirmed by more relevant prospective studies.

Consistent with previous findings grade, T stage, and M stage were identified as risk factors affecting patient prognosis (15–17). Among them, T stage and M stage showed a significant trend of worse prognosis with tumor progression, but Grade II showed a higher nomogram score than Grade III. We believe that this result may be due to the bias generated by the small sample size. Also of interest is that after Cox survival analysis, the N stage was excluded as a variable affecting the prognosis of patients with unoperated thyroid cancer (**Table 2**). Previously, it was well recognized that lymph node metastasis increased locoregional recurrence and mortality in thyroid cancer patients (13, 18). However, these are retrospective studies based on those who received surgery to remove the cancerous lesions. Therefore, we believe that for thyroid cancer patients who have not received surgery, whether lymph node metastasis occurs or not is no longer the main factor affecting the survival rate, and what should be emphasized more is whether distant metastasis occurs or not.

The priority of surgery for the management of non-inert thyroid cancer is indisputable (3). One study noted that almost all thyroid cancer patients in Korea underwent total or subtotal thyroidectomy (19). However, there is a lack of adequate clinical evidence for treatment strategies for patients who cannot undergo surgery due to medical conditions or financial reasons. In such cases, radiation therapy is usually the treatment of choice for non-operated patients. After PSM analysis to maximize the effect of confounding factors, a significant difference in survival prognosis between patients who received radiation therapy and those who did not (P<0.05) can be seen in **Figure 6**. This result suggests that radiation therapy for non-surgical thyroid cancer patients can still have a positive impact on long-term survival. Radioiodine therapy (RIT) is the most common treatment modality and has been used in clinical care for more than 60 years (20, 21). The treatment of papillary thyroid cancer with radiation therapy primarily involves external beam radiation therapy (EBRT) and radioisotope therapy (RAI), each with distinct applications. EBRT is primarily employed for local recurrence or residual disease, particularly when surgery or RAI is ineffective. It effectively controls local tumor growth and reduces recurrence, though it may cause side effects such as skin reactions and difficulty swallowing (22). In contrast, RAI is used postoperatively to ablate residual thyroid tissue and treat metastatic papillary thyroid cancer by administering radioactive iodine, which destroys cancer cells. This significantly reduces recurrence risk, though it may cause temporary side effects such as salivary gland swelling and dry mouth. In summary, EBRT is suitable for local treatment, while RAI offers systemic therapeutic effects (23). Both modalities can be used independently or in combination, depending on the specific clinical scenario, to achieve optimal treatment outcomes. For patients who cannot be surgically removed or for whom surgery is inappropriate, RIT can be used to control the growth of localized lesions, relieve symptoms, and improve patients’ quality of life (24–26). If there are metastases of cancer cells in the lymph node area using RIT may also control cancer cells in the lymph node area (27). However, in the treatment of low-risk thyroid cancer patients, the positive impact of RIT on long-term patient survival has not been demonstrated (21). Therefore, the results of our study may be able to serve as a reference for researchers to demonstrate the favorable role of radiation therapy in improving the prognosis of patients.

In addition, we believe that chemotherapy is also a potential risk factor that may affect the prognosis of patients with non-operated thyroid cancer. However, in the latest guidelines for the management of thyroid cancer, chemotherapy is still mainly used as an individualized treatment modality adjuvant to radiation therapy to enhance the anti-tumor effect (28, 29). In recent years, research on molecularly targeted therapies for thyroid cancer, such as tyrosine kinase inhibitors and anti-angiogenic drugs, is progressing at a rapid pace. These chemotherapeutic agents that act on cell proliferation, immunosuppression, and angiogenesis to inhibit tumors may become a new strategy for the treatment of thyroid cancer in the future (30).

## Conclusion

After Cox survival analysis, age, grade, T stage, M stage, radiotherapy, and chemotherapy were considered risk factors to construct a nomogram. We successfully developed and validated the nomogram that predicts CSS in patients with non-operated papillary thyroid cancer. After PSM analysis, radiotherapy was shown to have a positive effect on CSS in all patients with non-operated papillary thyroid cancer.

## Data Availability

The original contributions presented in the study are included in the article/supplementary material. Further inquiries can be directed to the corresponding author.
